# Recent Advances and Applications of Plant-Based Bioactive Saponins in Colloidal Multiphase Food Systems

**DOI:** 10.3390/molecules26196075

**Published:** 2021-10-08

**Authors:** Mengyue Xu, Zhili Wan, Xiaoquan Yang

**Affiliations:** 1 Laboratory of Food Proteins and Colloids, School of Food Science and Engineering, Guangdong Province Key Laboratory for Green Processing of Natural Products and Product Safety, South China University of Technology, Guangzhou 510640, China; xumengyue97@163.com (M.X.); fexqyang@scut.edu.cn (X.Y.); 2 Overseas Expertise Introduction Center for Discipline Innovation of Food Nutrition and Human Health (111 Center), Guangzhou 510640, China

**Keywords:** natural saponins, biosurfactants, glycyrrhizic acid, self-assembly, emulsions, foams, edible colloids

## Abstract

The naturally occurring saponins exhibit remarkable interfacial activity and also possess many biological activities linking to human health benefits, which make them particularly attractive as bifunctional building blocks for formulation of colloidal multiphase food systems. This review focuses on two commonly used food-grade saponins, Quillaja saponins (QS) and glycyrrhizic acid (GA), with the aim of clarifying the relationship between the structural features of saponin molecules and their subsequent self-assembly and interfacial properties. The recent applications of these two saponins in various colloidal multiphase systems, including liquid emulsions, gel emulsions, aqueous foams and complex emulsion foams, are then discussed. A particular emphasis is on the unique use of GA and GA nanofibrils as sole stabilizers for fabricating various multiphase food systems with many advanced qualities including simplicity, ultrastability, stimulability, structural viscoelasticity and processability. These natural saponin and saponin-based colloids are expected to be used as sustainable, plant-based ingredients for designing future foods, cosmetics and pharmaceuticals.

## 1. Introduction

Colloidal multiphase systems, such as emulsions and foams, are widely used in many fields, ranging from the formulations of food, personal care, cosmetic, detergent and pharmaceutical products to functional applications such as bioactive encapsulation and release, enhanced oil recovery or flotation [1,2,3,4]. Controlling the characteristic and properties of multiphase soft materials is crucial for their practical applications and also very challenging, since most of them are thermodynamically unstable systems and have high surface-area-to-volume ratios as well as structural complexity at multiple length scales [5,6,7]. Therefore, in practical processing, many types of surface-active components are added to reduce the energy at the interfaces of multiphase systems, forming the interfacial layers to stabilize dispersed oil droplets or air bubbles [2,3,8]. These stabilizers with surface activity used as are particularly important ingredients for producing commercial multiphase products with sufficient shelf-life stability and functional attributes.

For current industrial applications, many synthetic or semisynthetic surfactants, such as SDS, Tweens, Spans and sucrose esters, are widely used in most formulations of emulsifying and foaming agents. However, due to the increasing awareness of the importance of health and environmental sustainability, the demand for “clean label” products formulated with all-natural and sustainable ingredients has significantly increased, especially in foods and beverages [9,10,11]. For this reason, the naturally occurring surfactants (also called biosurfactants) with the nature of bioavailability, biocompatibility and biodegradability are highly desired by food manufacturers, who try to use them as natural alternatives for reformulating multiphase products. These natural surfactants, such as saponins, show interesting interfacial properties and often possess many biological activities for the human body, which make them have a great potential as bifunctional building blocks for formulations of multiphase food, cosmetic and pharmaceutical products [12,13,14].

Saponins are a class of natural phytochemicals, which can be found in more than 500 plant species [15]. They are usually extracted from many legumes, ginseng roots, licorice roots, spinach leaves, tea leaves and the bark of Quillaja saponaria Molina trees [15,16]. Generally, as a phytochemical, the content of saponins in plants is usually very low (about 1%), but the bark of Quillaja saponaria Molina tree contains up to 10% of saponins, which also is the main source of saponins (Quillaja saponins) for industrial applications [12,17]. The composition and saponin concentration of saponin extracts obviously depend on the plant species as well as the part of the plant, seasonal changes, the soil quality, extraction parameters and storage procedures, which have been extensively reported and reviewed in [12,13,15,18]. As secondary metabolites of plant metabolism, natural saponins also display a number of nontrivial biological activities, such as anti-inflammatory, antiviral, anticancer, hypolipidemic, cholesterol-lowering, adjuvant effects and free radical scavenging effects, which are currently under active investigation for many bio-related applications [15,16,19].

In recent years, as a class of natural surfactants, the saponins have attracted a lot of research interest due to their strong interfacial and biological activities, as well as the unique formation and stabilization of multiphase systems (e.g., emulsion and foams). The early studies mainly focused on Quillaja saponins, and their behaviors at the liquid interfaces (air–water and oil–water) including the adsorption kinetics and rheological properties of the adsorption layers have been systematically analyzed [20,21,22,23]. The effects of the molecular structure of saponins as well as the hydrophobic oil phase on the interfacial properties of these various saponins were also investigated [24]. In addition, the interactions of Quillaja saponins with other ingredients, such as food proteins [25,26], polysaccharides [27] and membrane lipids [28], in bulk solutions and at the air–water/oil–water interfaces were examined. The related applications of Quillaja saponins in multiphase food systems including emulsions (e.g., nanoemulsions, multiple emulsions and emulsion-templated oleogels), foams and lipid particles have also been extensively studied by many researchers [12,17,29,30,31,32]. Recent studies reported that another natural food-grade saponin glycyrrhizic acid (GA) can form novel saponin nanofibrils through a unique fibrillar self-assembly behavior in aqueous solutions, and these assembled GA nanofibrils can be used as a sole building block to fabricate stable emulsions, foams and even complex emulsion foams [33,34,35,36]. These recent literatures well demonstrate the rich self-assembly and interfacial properties of natural saponins and their promising applications in various multiphase food systems.

Herein, we will provide an overview of the recent literatures available on the interfacial behaviors of natural saponins at air–water and oil–water interfaces and their applications in different colloidal multiphase food systems, such as emulsions, gel emulsions, aqueous foams and complex emulsion foams. Considering the structural diversity of saponins and its impact on the self-assembly and interfacial properties, we intend to focus this review mainly on Quillaja saponins (QS) and glycyrrhizic acid (GA), with the aim of clarifying the relationship between structural features of saponin molecules and their subsequent self-assembly and interfacial properties. QS and GA are also two food-grade saponins that have been approved as food additives for common use in the food industry. Finally, we will discuss the applications of natural saponins in different colloidal multiphase systems and the interactions with other components in more complex systems. We expect the natural saponin and saponin-based colloids can be used as sustainable, plant-based ingredients for designing future foods, cosmetics and pharmaceuticals.

## 2. Biological Activities of Saponins

Saponins exhibit a wide range of biological activities, such as hypocholesterolemic, hypoglycemic, hepatoprotective, hemolytic, immunomodulatory, antiviral, anti-inflammatory and antitumor activities [15,16]. Many of these activities are usually associated with the interactions of saponins with the lipids of biological membranes, such as cholesterol and phospholipids [37,38,39,40,41,42]. Numerous studies have demonstrated the ability of saponins to inhibit cholesterol absorption and to decrease serum and liver cholesterol [43,44]. Vinarova et al. recently studied the effect of six saponin extracts on the bioaccessibility of cholesterol and saturated fatty acids by using in vitro digestion model and in vivo animal studies [37,38]. They found that the saponin extracts (Quillaja Dry and Sapindin) can decrease cholesterol bioaccessibility up to 44%, and the main mechanism of cholesterol-lowering effect is the displacement of cholesterol from the dietary mixed micelles, which can lead to the direct precipitation of cholesterol that cannot pass through the mucus layer of the intestine. As one of the most common saponins, Quillaja saponins (QS) are known to interact with biological membranes, and the model DPPC monolayer studies showed that they can effectively penetrate phospholipid mono- and bilayers and, meanwhile, do not disrupt DPPC monolayers [39]. Sosnowski et al. found that QS may be useful in biophysical studies related to pulmonary surfactant dynamics, since it has the ability of enhancing the phospholipid mass exchange between the interface and the liquid subphase [42]. Previous studies have shown that saponins (e.g., QS) can be wrapped by neutral phospholipids and cholesterol as an immunostimulating complex (ISCOM), which can effectively enhance humoral and cellular immune responses [45,46] and thus make the saponins have the ability to be used as appropriate vaccine adjuvants. In addition, the dietary saponins can exert the nutritional effects by decreasing the synthesis of lipids, suppressing adipogenesis and inhibiting intestinal absorption of lipids, which may help in protecting against the development of obesity [47].

The antivirus activities of saponins have attracted increasing attention in recent years. Sharma et al. have reviewed the advancements on the antiviral activity of saponins against various viruses [48]. Particularly, glycyrrhizic acid (GA) and its derivatives are shown to have remarkable antiviral properties and can inhibit the infection of a variety of viruses, such as SARS-associated human and animal coronaviruses, dengue virus, vaccinia virus, duck hepatitis virus, HIV-1 virus, infectious hepatitis C virus (HCV) and human respiratory syncytial virus (HRSV) [19,49,50,51]. Coronavirus can cause a disease with high infectivity and pathogenicity, especially SARS in 2003, MERS in 2012, and COVID-19 currently. In 2003, it was reported that GA has the ability to effectively inhibit the replication of two clinical isolates of SARS-associated coronavirus (SARS-CoV, FFM-1 and FFM-2) [52]. Recently, many studies have demonstrated that the GA can be considered as one promising anti-SARS-CoV-2 drug candidate, alone and in combination with other drugs (e.g., chloroquine and tenofovir), to combat the current COVID-19 pandemic [19]. This is mainly due to the capacity of GA to bind to the angiotensin converting enzyme 2 (ACE2), a SARS-CoV-2 receptor, which can prevent the virus from diffusing out of infected cells and to enter new cells [19,53,54,55]. In addition, other structurally related saponins with the membrane-perturbating effects, such as escin, platycodin D and saikosaponin, also exhibit strong biological activity against coronavirus [19,56,57].

## 3. Molecular Structure and Self-Assembly in Aqueous Solutions

**Figure 1:**
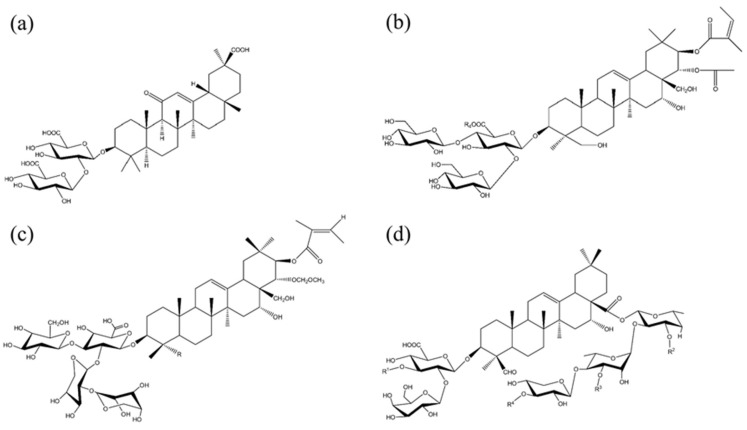
Molecular structures of three monodesmosidic saponins, glycyrrhizic acid (**a**), escin (**b**) and tea saponin (**c**), and bidesmosidic saponin Quillaja saponin (**d**).

Saponin molecules have a typical amphiphilic structure consisting of a hydrophobic triterpenoid or steroid backbone (aglycone) and one or more hydrophilic oligosaccharides (sugar), which are attached to the aglycone via glycoside bonds [15,58]. The classification of saponins is mostly based on the type of aglycone structure, which is either a triterpenoid, steroid or steroid–alkaloid group, and the number of linked sugar chains, which may be mono-, di- or even tridesmosidic, comprising one, two or three sugar groups, respectively [12,13,14]. The common sugar groups of saccharide chain commonly include, glucose, galactose, rhamnose, arabinose, xylose, fructose and glucuronic acid. The chemical structures of four commonly studied saponins, including Quillaja saponin (QS), escin (ESC), tea saponin (TS) and glycyrrhizic acid (GA), are shown in Figure 1. As can be seen, the TS, ESC and GA are monodesmosidic saponins, whereas the QS is bidesmosidic saponin. Currently, the commercially available QS products are almost exclusively extracted from the bark of the tree Quillaja saponaria Molina, and the purity, composition and functionality of QS extracts vary with different suppliers and their extraction procedures [17]. GA is extracted from the root of the licorice plants (Glycyrrhiza glabra), and the commercial products generally have high purity (above 95%). The diversity of amphiphilic structures of the saponins, depending upon the different plant species of origin, determines their rich physicochemical properties (e.g., self-assembly behaviors) and different biological activities and applications.

### 3.1. Saponin Micelles

The amphiphilic structure of saponin molecules determine their self-assembly behaviors in aqueous solutions. Many saponins have been demonstrated to have micelle-forming properties, and the saponin molecules can aggregate into micelles above a critical micelle concentration (CMC). The CMC is also commonly used parameter for the comparison of the interfacial activities of various saponins, and the lower CMC values generally represent the higher surface activity. The CMC is very different between various saponins and saponin extracts, which can be affected by the molecular structure, plant species and even commercial sources of saponins. For example, the CMC values for Quillaja saponin (QS) provided by different suppliers (Sigma, Desert King, Acros Organics and Penco of Lyndhurst) can be ranged from 0.01 g/L up to 0.77 g/L [23,59,60]. The spherical micelles of QS formed in aqueous solutions are reported to have a hydrodynamic radius of around 3.6 nm, and the aggregation number of micelles is around 49 [59,60]. The changes of temperature, pH and salt concentration are demonstrated to affect the micellar properties of QS solutions, such as the CMC, size and intrinsic viscosity of micelles [59]. Recently, Tippel et al. investigated the solubilization of poorly water-soluble lutein ester in aqueous solutions of QS micelles and found that the lutein ester-loaded QS micelles showed a significantly larger particle size, and the micelle diameter can reach up to 130 nm [60]. In addition, they observed an elongated/worm-like structure for the lutein ester-loaded QS micelles at pH 3, which is explained by the fact that the decreased electrostatic repulsion between the charged headgroups leads to an increase in the critical packing parameter, thus causing the elongation of QS micelles [60].

### 3.2. Glycyrrhizic Acid Nanofibrils and Supramolecular Hydrogels

**Figure 2:**
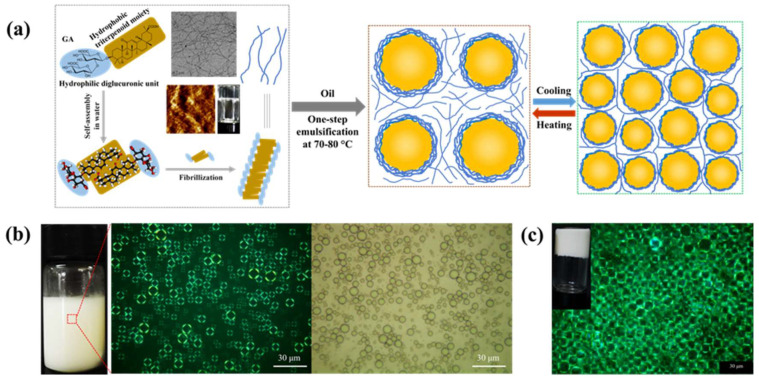
(**a**) Schematic illustration of the fibrillar self-assembly of glycyrrhizic acid (GA) molecules in water, forming GA nanofibrils and hydrogels, and of the formation of thermoresponsive gel emulsions by the multilayer adsorption of GA nanofibrils at the oil–water interface and the assembly of fibrillar hydrogel network in the continuous phase after cooling. (**b**) PLM (left) and optical microscopy (right) images of the reconstituted emulsions with 0.25 wt% GA nanofibrils from the gel emulsions stabilized by 4 wt% GA nanofibrils. The radiant halo with a Maltese cross in PLM image reveals the multilayer fibril shell structure of the droplets. (**c**) Photograph and PLM image of the gel emulsion with 60 wt% olive oil prepared using 4 wt% GA nanofibrils. Images were reproduced with permission from [33].

Glycyrrhizic acid (GA) is a monodesmosidic triterpenoid saponin derived from the licorice root. GA solutions are overall stable to the exposure of base, neutral, oxidation and heating treatment; however, it is important to avoid excessive heating to prevent conversion into glycyrrhetinic acid. The chemical structure of GA molecules consists of a hydrophobic triterpenoid aglycon moiety (18β-glycyrrhetinic acid) attached to a hydrophilic diglucuronic unit (Figure 1a). Due to the presence of spatial configuration in C18, the natural GA exists as two epimers 18α-GA and 18β-GA, and the latter seems to have stronger biological and surface activities and is more commonly used in research and practical applications. Owing to the amphiphilic structure and chirality, GA molecules exhibit a hierarchical self-assembly behavior in water [33,61], which is different from the aqueous solutions of other triterpenoid saponins. The self-assembly of GA in water strongly depends on the concentration of GA, and the gradual increase in concentration of GA molecules leads to the formation of long nanofibrils first, which then form a fibrillar network upon increasing concentrations and finally form a nematic supramolecular hydrogel when the nanofibril concentration is above 0.3 wt% [33,61]. Through the combination of small-angle X-ray scattering and atomic force microscopy, Saha et al. demonstrated that the assembled GA nanofibrils in water are long and semiflexible with the right-handed twist, 2.5 nm thickness and 9 nm periodicity, independently of GA concentration [61]. They suggested the formation of GA nanofibrils is due to the lateral interactions between the hydrophobic triterpenoid moieties of GA molecules, yielding a head-to-head configuration and leaving the hydrophilic sugar groups exposed to water (Figure 2a). In addition, the supramolecular GA hydrogels are thermoresponsive with a gel–sol transition temperature around 55–60 °C [33,62], and above this temperature, the fibrillar network initiates melting due to the reduced interfibrillar hydrogen bonding. Recent study also showed that natural GA hydrogels exhibited great injectable and moldable properties and also have inherent antibacterial ability to effectively inhibit the growth of Gram-positive *Staphylococcus aureus*, suggesting their potential application in biomaterials and 3D bioprinting [63].

GA is a polyprotic weak acid with three carboxylic groups, and thus, it has three dissociation constant values, which are 3.98 (pK_a1_), 4.62 (pK_a2_) and 5.17 (pK_a3_) [64]. The degree of dissociation of three carboxylic groups under different pH values is shown to strongly affect the solubility and self-assembly of GA molecules in aqueous solutions. The low degree of dissociation of the carbonyl groups at low pH values leads to a lower solubility of GA. Accordingly, at pH > 5.5, the carbonyl groups are expected to be fully dissociated, and the solubility is, thus, higher. It has been demonstrated that the degree of dissociation of carboxylic groups also affects the self-assembly of GA molecules forming different structures, such as micelles, nanofibrils and hydrogels [33,61,65,66]. It can be confirmed that, within the pH range of 2–5, GA molecules in water can self-assemble into nanofibrils and then form hydrogels through interfibrillar hydrogen-bond interactions. In contrast, when the pH values are higher than 5, there is no gel formation for GA water solutions. Matsuoka et al. reported that the GA formed rod-like micelles at pH 5–6 [65], whereas at pH 7 or higher values, there is no detectable micellar structure, and the GA should exist as monomers in solutions [65,66].

## 4. Behaviors of Saponin Molecules and Assemblies at Liquid Interfaces

Saponin molecules have strong surface activity due to their amphiphilic structure. The interfacial behaviors of saponins at the liquid interfaces (air–water and oil–water) often play a crucial role in determining their use as emulsifiers and foaming agents for practical applications. Previous studies have demonstrated that the molecular structure of saponins and the hydrophobicity of the oil phase can affect the adsorption kinetics and rheological properties of the saponin adsorption layers [22,23,24]. In addition, a more detailed understanding of the relation between interfacial and self-assembly properties of saponins is required. These factors are closely related with the applications of saponins in multiphase food systems.

### 4.1. Interfacial Properties and Configuration of Saponin Molecules

The interfacial behaviors of various saponins including the adsorption and rheological properties of the saponin adsorption layer have attracted a lot of research interest in recent years [12,13,20,21,22,23,24]. The obtained results showed that, apart from the superior surface activity, the adsorption layers stabilized by saponins exhibit unusual surface rheological properties, such as remarkably high surface dilatational and shear elasticities, and upon expansion and compression, these layers display nonlinear rheological responses, even at relatively small deformations [20,21,22,67]. The surface rheological properties of the adsorption layer from Quillaja saponins (QS) have been first studied in depth [20,21], and the obtained results showed that the QS-stabilized interfacial layers exhibit a very high surface dilatational elasticity (up to 280 mN/m), a negligible dilatational viscosity and noticeable shear elasticity. Additionally, the viscoelastic response of the QS adsorption layer is sensitive to shear stress, and high shear stress can lead to the disruption of the internal solid structure of the adsorption layer. Tcholakova and coworkers then investigated the surface rheological properties of adsorption layers both in dilatation and in shear deformation formed from a range of triterpenoid and steroidal saponins [22,67]. They found that most of the triterpenoid saponins including ESC, TS and Berry saponins exhibit complex viscoelastic properties with extremely high elastic modulus (under both shear and dilatational deformations) and viscosity. These three saponins all contain mainly monodesmosidic triterpenoid saponins, and their viscoelastic properties can be explained by the strong attractions between the adsorbed saponin molecules, which probably arise from the multiple hydrogen bonds between the neighboring sugar groups of the saponin molecules in the densely packed adsorption layers. In contrast, the adsorption layers of all steroid saponins have no shear and dilatational elastic properties and very low surface viscosity, which is due to the fact that they cannot form strong intermolecular bonds at the interface [22,67]. On the basis of the abovementioned results, it is clear that the molecular structure of the various saponins has an important impact on the surface rheological properties of saponin adsorption layers.

The molecular structure of various saponins including the type of their aglycone (triterpenoid or steroid) and the number of sugar chains (1–3) can also strongly affect the configuration behaviors of saponins at the interface. The values of the area per molecule in equilibrium saponin adsorption layers, determined from the surface tension isotherms at the air–water interface, have the meaning of geometrical orientation of the molecules at the interface. For saponins, if the value of the area per molecule is lower than 0.75 nm^2^, it means an orientation of the molecules with the side-on/end-on interfacial configurations, whereas an area per molecule higher than 0.75 nm^2^ corresponds to the lay-on configuration [20,67]. The side-on configuration describes the orientation of monodesmosidic triterpenoid saponins where the aglycones are located parallel to each other in hydrophobic air phase and perpendicular to the interface. Bidesmosidic saponins are mostly oriented in the end-on and lay-on configurations. The former is similar to the side-on configuration, but there is one hydrophilic sugar chain in the hydrophobic phase besides the aglycone. In the lay-on configuration, both hydrophilic sugar chains are located into the water phase, and the aglycone lays parallel to the interface. Pagureva et al. reported that the monodesmosidic triterpenoid saponins ESC and TS have area per molecule in the range between 0.5 and 0.7 nm^2^ and, thus, they are orientated perpendicularly to the interface [67]. Bidesmosidic triterpenoid saponins are probably oriented in the lay-on configuration with an area per molecule of around 1 nm^2^ [20,67]. However, for the bidesmosidic saponin QS, it remains unclear if they are always orientated in the lay-on configuration at the air–water interface, since the QS from different extracts are reported have different values of area per molecule, in the range of 0.4–1.2 nm^2^ [20,23,67,68]. In addition, the bidesmosidic saponin Ginsenosides have an area per molecule of around 0.5 nm^2^, which means that they are located approximately perpendicular to the interface with the end-on configuration.

### 4.2. Interfacial Behaviors of Glycyrrhizic Acid Nanofibrils

Golemanov et al. analyzed the adsorption and shear rheological properties of glycyrrhizic acid (GA) molecules at the air–water interface, and they found that GA probably forms nonelastic layers with low viscosity [22]. They attributed the surface properties to the very low solubility of GA in water, thus leading to the insufficient adsorption of GA molecules to form a packed interfacial layer. Recently, Wan et al. found that, compared to the GA monomers, the GA nanofibrils, obtained from supramolecular self-assembly of GA molecules in water, exhibit significantly different behaviors at the liquid interfaces [33,34,35,36]. First, it should be noted that the long, semiflexible GA nanofibrils are structurally similar to the amyloid fibrils from heat-induced self-assembly of globular food proteins, such as whey proteins and soy proteins [69,70,71]. Protein-based nanofibrils have been shown to have satisfactory surface activity and are able to stabilize the liquid interfaces (air–water and oil–water) efficiently [71,72,73,74]. As expected, considering the structural similarity, the GA nanofibrils show some similar interfacial behaviors to protein fibrils, such as the multilayer adsorption at the interface. On the other hand, another type of rod-like polymer-based nanostructures such as cellulose nanofibrils or nanocrystals, which have high aspect ratio with a few nanometers in width and several micrometers in length, are also demonstrated to be efficient Pickering-type interfacial stabilizers for preparing foams and emulsions [75,76,77]. Compared to these short and rigid polymer nanorods, the ultrafine, semiflexible GA nanofibrils (2.5 nm in diameter) possess higher aspect ratio, homogeneous wettability and favorable interfacial activity, which make them more suitable for the stabilization of the curved soft interfaces, including the air–water and oil–water interfaces. As a result, the GA nanofibrils exhibit the ability to rapidly adsorb at the interfaces and reduce the interfacial tension, which endow them with a high foaming and emulsifying capacities [33,34,35,36,78,79]. The multilayer assembly behavior of GA nanofibrils at the interface can lead to the formation of a dense interfacial fibril network (Figure 2a,b), which can provide the GA nanofibril-stabilized emulsion droplets and air bubbles with a fully covered surface [33,34,35,36]. Wan et al. demonstrated that such interfacial fibril network with a high electrostatic force can provide a superior stability to the GA nanofibril-covered emulsion droplets during heating at a high temperature (80 °C, 20 min) and storage at room temperature for 60 days [33]. Recently, Ma et al. reported that the GA nanofibril water solutions can form viscoelastic adsorption layer at the n-tetradecane–water interface, and the dilatational storage modulus can reach 18.2 mN/m at low GA concentration of 0.01 wt% [80].

## 5. Applications of Saponins in Colloidal Multiphase Systems

The formation and stabilization of colloidal multiphase systems such as emulsions and foams require the addition of surface-active materials, which can reduce the high interfacial energy of systems and form the adsorption layers to stabilize dispersed oil droplets or air bubbles. As mentioned above, many saponins have strong surface activity and can form the adsorption layers with high elastic properties. These surface properties are important for the applications of saponins in colloidal multiphase systems. In this section, we will focus on the discussion of the applications of two commonly used food-grade saponins, Quillaja saponins (QS) and glycyrrhizic acid (GA) in various multiphase food systems, including liquid emulsions, gel emulsions, aqueous foams and complex emulsion foams (Table 1 and Table 2).

### 5.1. Liquid Emulsions

**Figure 3:**
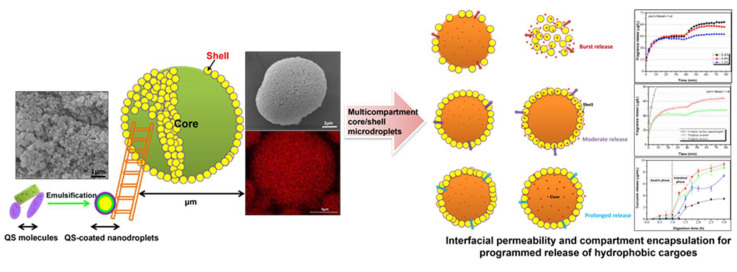
Schematic illustration of the mechanism for programmed release of hydrophobic cargoes from the multicompartment microdroplets stabilized by Quillaja saponin (QS)-coated nanodroplets. Images were reproduced with permission from [87].

Due to the strong interfacial activity and the relatively low molecular weight, many saponins can rapidly reduce the interfacial tension and pack efficiently at the oil–water interface [23,26,29]. The equilibrium interfacial tension of Quillaja saponins (QS) can reach 5.0 mN/m at the medium-chain triglyceride–water interface, which makes QS have good emulsifying activity and the ability of forming small oil droplets (nanoemulsions) [29,30,81]. The emulsions stabilized by QS (Q-Naturale^®^) can remain stable against droplet coalescence under varying pH values (2–8), ionic strengths (0–500 mM NaCl), temperatures (20–90 °C) and after long-term storage at room temperature [29,82,83]. However, very low pH value (pH 2) and high salt concentration (above 400 mM NaCl) could lead to the droplet flocculation and creaming, which is due to the screening of charged groups and thus the reduced electrostatic repulsion between the QS-covered droplets under these conditions [29]. The emulsion and nanoemulsions stabilized by QS exhibit multifunctional properties and applications. For example, the QS-based nanoemulsions can be used as stable delivery systems for hydrophobic bioactive substances, improving their solubility and bioavailability [30,84]. Moreover, the QS extracts can provide positive effects on the inhibition of lipid oxidation in in oil-in-water emulsions [85], and the emulsions also can be used to control flavor retention and release during simulated cooking [86]. Chen et al. recently reported that the nanodroplets stabilized by QS can further serve as building blocks for fabricating microscale emulsion droplets, which display multicompartment architectures comprised of many nanodroplets as an interfacial shell and a single microdroplet core (Figure 3) [87]. They found that the prepared multicompartment emulsion droplets can allow the programmed release of various volatile compounds by controlling the number of the QS-based nanodroplets around the surfaces of microdroplets, which can accurately manipulate the interfacial permeability (Figure 3).

Recently, another natural saponin glycyrrhizic acid (GA) and the assembled GA nanofibrils have been shown to be efficient natural emulsifiers for formation and stabilization of food emulsions [33,88]. Wan et al. found that the amphiphilic GA monomers and assembled nanofibrils have high affinity toward the hydrophobic oil phase and, thus, can decrease the interfacial tension effectively, which provides the GA and GA nanofibrils with satisfactory emulsifying properties [33]. The oil-in-water emulsions prepared by GA nanofibrils (0.25 wt%) showed homogeneous size distribution and can remain stable after repeated heat treatments (80 °C, 20 min) and storage at room temperature for two months. They attributed the superior stability of emulsions to the formation of multilayer GA fibril shells with a high electrostatic repulsive force (Figure 2), which, thus, effectively protect the emulsion droplets against flocculation and coalescence. They further found that the oil polarity had a significant impact on the interfacial and emulsion properties of GA nanofibrils. For more polar oils (e.g., algal oil), the GA nanofibrils exhibited a higher affinity to the oil–water interface and, thus, a faster adsorption kinetics, which led to the formation of multilayer emulsion droplets with smaller droplet size [33]. Weiss and coworkers recently investigated the emulsifying properties of GA monomers at pH 7.0 and showed that the GA (0.01–5 mM) can form stable oil-in-water emulsions (10 wt% oil) with a d_32_ value of around 0.2 μm [89]. These emulsions are found to have good stability under various environmental stresses, including a wide range of pH values (5–9), salt concentrations (0–200 mM NaCl) and temperatures (up to 60 °C) [90].

### 5.2. Gel Emulsions

Gel emulsions (or called emulsion gels) are a type of complex soft-solid emulsion materials with both the properties of traditional multiphase emulsions and the behaviors of physical gels, in which the oil droplets are entrapped within a gel matrix. As one of the most common type of gel emulsions, high internal phase emulsions (HIPEs) that possess an internal phase volume fraction exceeding 0.74 have shown great potentials in various applications. Chen et al. reported that the Quillaja saponin (QS)-coated nanodroplets can be used as stabilizers for the fabrication of stable oil-in-water HIPEs (75% oil), and the obtained HIPEs can be further converted into transparent oleogels (99.7% oil) through oven drying (70 °C) [31]. Overall, the solid-like viscoelastic behaviors of the HIPEs mainly rely on the tight stacking of the dispersed oil droplets within the matrix.

**Figure 4:**
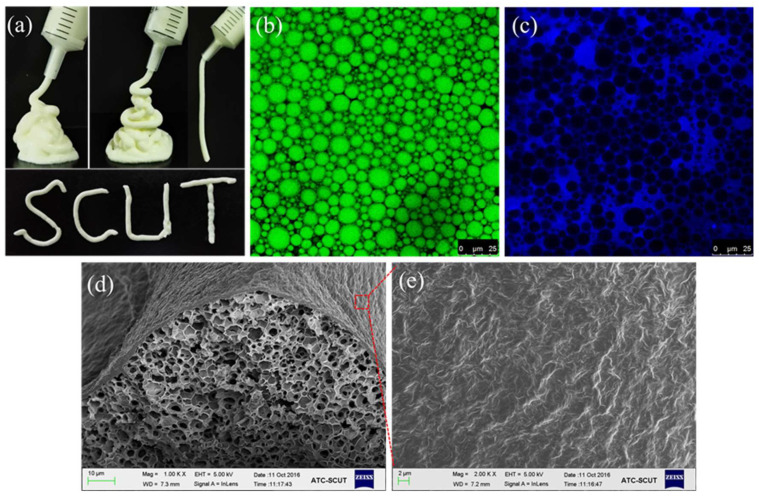
(**a**) Photographs of the gel emulsions (60 wt% olive oil, 2–4 wt% GA nanofibrils) with the shape of alphabets using a plastic syringe after cooling the extruded warm emulsions at room temperature (25 °C) for 1 min. (**b**,**c**) CLSM images of gel emulsions with 60 wt% olive oil prepared using 4 wt% GA nanofibrils. The sample was prepared with the oil dyed with Nile Red (**b**) and the GA fibrillar network dyed with ThT (**c**), respectively. (**d**,**e**) SEM images of 4 wt% GA nanofibril gel emulsion with 40 wt% hexane as oil phase. The sample was prepared at air-drying condition. Images were reproduced with permission from [33,34].

Recently, Wan et al. firstly reported that the food-grade saponin glycyrrhizic acid (GA) and GA nanofibrils can be used as a sole stabilizer to make novel oil-in-water gel emulsions (Figure 2 and Figure 4) [33]. Through a facile one-step emulsification at high temperature (80 °C) followed by a subsequent cooling, they successfully prepared stable gel emulsions with many interesting rheological behaviors, such as high gel strength and good ability of thixotropic recovery. The formation and stabilization of gel emulsions is mainly attributed to the spatially controllable self-assembly of GA nanofibrils at the oil–water interface and in the aqueous phase, which forms the multilayer fibril shells around the oil droplets and the viscoelastic hydrogel networks in the continuous phase to entrap the droplets (Figure 2a and Figure 4b–e). The gel emulsions also have interesting temperature-responsive behavior due to the presence of the thermoreversible gel–sol transitions of the fibrillar network in the continuous phase, and by simply changing the temperature (below and above 55–60 °C), the emulsion gels can be switched reversibly between a gel and liquid (sol) state (Figure 2a) [33]. Furthermore, they further found that the oil phase polarity can tune the microstructure and mechanical properties of these gel emulsion gels, and the emulsion gels with more polar algal oil showed a denser network microstructure and higher mechanical strength, which is due to the more compact packing of smaller emulsion droplets and, thus, the stronger interdroplet interactions within the network matrix [34]. Ma et al. also used the GA nanofibrils as stabilizer to fabricate stable gel-like emulsions with different agricultural oils for developing ecofriendly pesticide formulations [91].

The combined use of GA nanofibrils and the common lipophilic emulsifier polyglycerol polyricinoleate (PGPR) can allow the development of a gelled multiple water-in-oil-in-water (W_1_/O/W_2_) emulsion, which exhibited relatively homogeneous size distribution, high yield (85.6–92.5%) and superior storage stability [78]. The highly viscoelastic GA hydrogel in the continuous phase is found to play an important role in preventing the osmotic-driven water diffusion from the internal water droplets to the external water phase of W_1_/O/W_2_ emulsions. Recent study showed that the combination of GA nanofibrils and other structuring components is an effective strategy for fabricating structured gel emulsions with a more complex microstructure and more diverse rheological properties [92]. By controlling the self-assembly of GA nanofibrils and sitosterol–oryzanol system in aqueous solutions and oil phase, respectively, dual-structured gel emulsions with heterogeneous microstructures including a percolated segregated network and jamming transition were obtained, which showed different linear and nonlinear viscoelastic behaviors. The large amplitude oscillatory shear (LAOS) rheology showed that, compared to the jammed emulsion gel, the percolating emulsion gels had higher structural elasticity and, thus, were more resistant to large deformations [92]. In addition, the incorporation of colloidal nanoparticles can also tune the formation, microstructure and mechanical properties of emulsion gels stabilized by GA nanofibrils by the nanofibril–nanoparticle interactions. Li et al. showed that, for the emulsion systems made by the mixtures of GA nanofibrils and the soy-protein-isolate–pectin complex nanoparticles, at low nanofibril concentrations (<0.5 wt%), obvious flocculation and clustering of oil droplets induced by a depletion mechanism were observed in the emulsions, whereas the emulsion gels with small droplet size, homogeneous appearance and microstructure can be obtained when the GA nanofibril concentration is above 1 wt% [88]. Qiu et al. also reported that, compared to the individual building block, the mixtures of nanocyclodextrin-based metal–organic frameworks (CD–MOF) and GA nanofibrils at the mass ratio of 2:5 showed a relatively lower interfacial tension and had the better ability to produce emulsions and emulsion gels with long-term stability, even under high-alkaline pH and high-temperature (70 °C) [93].

### 5.3. Aqueous Foams

Although the adsorption and surface rheological properties of the Quillaja saponins (QS) at the air–water interface have been systematically investigated, which are thought to be closely related the foamability and foam stability, the number of studies about the foaming behavior of QS and its relation with the surface properties is still relatively limited [26,68,94,95,96]. Wojciechowski and coworkers investigated the relation between the adsorption dynamics and the foamability of QS and the QS–protein mixtures [26,94]. They found that the synergistic effects in interfacial adsorption of QS–protein mixtures are much less noticeable in the foamability of systems, and the presence of proteins (β-lactoglobulin and lysozyme) can slightly enhance the foaming properties of QS, especially at low QS concentrations (<0.05 mM). Böttcher and Drusch studied and compared the foam properties and bubble structure of different saponin extracts from various sources [68]. Two extracts from Quillaja saponaria Molina (QS) and Gypsophila (bidesmosidic saponins) and two extracts from Camellia oleifera Abel (TS) and Aesculus hippocastanum (ESC) (monodesmosidic saponins) were found to be able to make stable foams. This is thought to be attributed to their rapid adsorption at the air–water interface and the formation of saponin adsorption layers with high dilatational and shear viscoelasticity [22,67]. In addition, the changes in pH values (3–5) and salt concentrations (up to 500 mM NaCl) only slightly affected the QS-stabilized foams [68]. Recent study also reported that the use of saponins as stabilizers can significantly reduce the rate of (or coarsening) in aqueous foams [95]. Through the theoretical predictions and the experimental data, Tcholakova et al. showed that the reduced rate of Ostwald ripening in saponin-stabilized foams is mainly due to the high resistance to gas transfer of the saponin adsorption layers [95].

**Figure 5:**
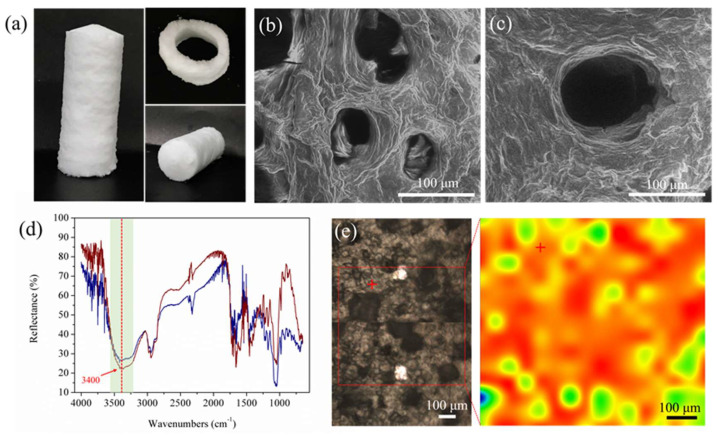
(**a**) Photographs of initial aqueous foam stabilized by 8 wt% GA nanofibrils, obtained by cooling in an ice bath (2 °C), made with cylindrical and annular molds. (**b**,**c**) Environmental SEM images of this initial wet foam. (**d**) FTIR spectra collected from this foam in D_2_O and (**e**) FTIR image (right) and the corresponding bright field image (left) of this foam, obtained by plotting the reflectance at 3400 cm^−1^ (associated with hydrogen bonding); colors qualitatively indicate the strength gradients of hydrogen bonds from low (blue) to high (red), showing the strong interfibrillar hydrogen bonding in the continuous phase as well as around the bubble surfaces. Images were reproduced with permission from [35].

Due to the thermodynamic instability as well as the structural complexity at multiple length scales, the fabrication of highly stable aqueous foams with a good foamability remains a big challenge in foam science. Wan and coworkers found that the saponin glycyrrhizic acid (GA) can be used as a sole stabilizer to make ultrastable aqueous foams, which can remain intact with a homogeneous appearance for at least six months at room temperature (25 °C) (Figure 5) [35]. The formation of this food-grade “superfoam” is attributed to the spatially controllable self-assembly of GA nanofibrils at the air–water interface and in the continuous phase, which form the multilayer interfacial network around the bubbles and the viscoelastic fibrillar hydrogel networks in bulk matrix (Figure 5b,c), respectively. Both of them provide the foams (above 4 wt% GA nanofibril) with the ultrastability over months or years without any detectable liquid drainage, and such long lifetimes are unprecedented in the foams prepared from the common food-grade foaming agents such as proteins, polysaccharides, small surfactants or their mixtures [2,3]. These ultrastable foams are thermoresponsive and can be rapidly destabilized by heating, which induces the melting of the hydrogen-bonded hydrogel networks inside the foams (Figure 5d,e). This is the first report on the use of a natural edible saponin GA as a sole stabilizer to make aqueous foams that have many advanced qualities including simplicity, high foamability, ultrastability, stimulability and processability. More recently, Wan and coworkers further explored the impact of the incorporation of rigid nanofiller cellulose nanocrystals (CNCs) on the structure and diverse properties including rheological properties, stability and stimuli responsiveness of the aqueous gel foams made by GA nanofibrils (4 wt%) [79]. CNCs were found to homogeneously distributed in the matrix of gel foams and provide the composite hydrogel network in the continuous phase with higher elastic modulus and yield stress (especially in the presence of NaCl). The composite gel foams prepared by the synergistic combination of semiflexible GA nanofibrils with rigid CNCs displayed tunable rheological properties, stability and thermoresponsive behavior [79].

### 5.4. Complex Emulsion Foams

In the area of soft condensed matter, there is a specific system containing both oil and air dispersed in an aqueous matrix, leading to the coexistence of oil droplets and air bubbles. These complex systems can be considered either as emulsion foams (called foamed emulsions) or as a specific aqueous foam whose interstitial fluid is doped with oil droplets [75,97]. Chen et al. reported that the Quillaja saponin (QS)-coated nanosized emulsion droplets (about 150 nm) can be used as Pickering-type interfacial stabilizers to fabricate aqueous foams [98]. The QS-coated nanodroplets showed a strong attachment at the air–water interface and were also entrapped and well-distributed in the foam liquid channels, which contribute to the significantly enhanced foamability and foam stability, as compared to those foams stabilized by QS. In addition, the QS nanodroplet-stabilized foam systems also exhibited the capacity for encapsulation and controlled release of hydrophobic flavors and bioactives (e.g., β-carotene and curcumin).

**Figure 6:**
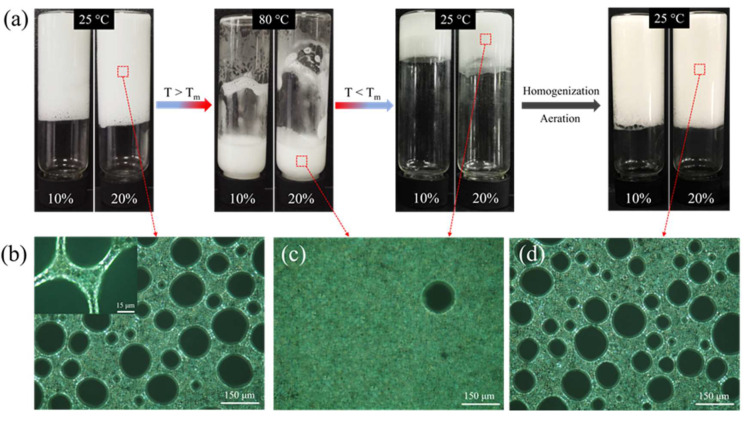
(**a**) Photographs showing the temperature-switchable process for complex emulsion foams containing 10 and 20 wt% oils stabilized by 4 wt% GA nanofibrils. Stable emulsion foams can again be obtained after homogenizing and aerating the mixtures (**c**) at 80 °C followed by cooling in an ice bath. (**b**–**d**) PLM images of the above samples with 20 wt% oil during the temperature-switchable process. Inset PLM image shows the jamming of the oil droplets in the liquid channels as well as around the bubbles. Images were reproduced with permission from [36].

For the stabilization of complex emulsion foams, it is generally required that the stabilizers can either form a stable interfacial layer at both the air–water and oil–water interfaces or form the gel network in the foam liquid films or both [5,36]. As discussed above, the glycyrrhizic acid (GA) and GA nanofibrils possess the multiple unique properties of satisfactory emulsifying, foaming and gelation abilities. Therefore, Wan et al. suggested that the GA nanofibrils can be used as a sole stabilizer to directly fabricate complex emulsion foams (Figure 6) [36]. They showed that, through one-step aeration at high temperature followed by rapid cooling in an ice bath, an emulsion foam that can be stable for at least two weeks was obtained, and the high foam stability is mainly attributed to the highly viscoelastic hydrogel networks in bulk phase, as well as the jamming of the emulsion droplets in the liquid channels (Figure 6b). These emulsion foams stabilized by GA nanofibrils are also thermoresponsive (Figure 6a), and it is reported that the bubble structure in the system completely disappeared in only 5 min at 80 °C [36]. They further developed dual photo-/thermoresponsive emulsion foams by a simple combination of the GA nanofibrils with the carbon black particles (CBP), which can absorb UV light and convert the absorbed light energy into heat. Accordingly, the stability and on-demand destabilization of emulsion foams can be better controlled by multiple external stimuli, such as temperature and light [36].

**Table 1:** Overview of applications of Quillaja saponin (QS) in colloidal multiphase food systems.

Type of Multiphase Systems	Structural Building Blocks	Properties and Applications	Ref.
Liquid O/W emulsions	QS molecules	Properties: Nanoemulsions; high stability under pH 2–8, 0–500 mM NaCl, temperatures of 20–90 °C and after long-term storage. Applications: Delivery systems for hydrophobic bioactives; Inhibition of lipid oxidation; Controlled flavor retention and release during simulated cooking.	[29,82,83,84,85,86]
	QS-coated nanodroplets	Properties: Multicompartment shell comprising nanodroplets; Good stability under pH values 3–7, salts 0–500 mM NaCl and temperatures 25–100 °C. Applications: Programmed release of volatiles.	[87]
Gel emulsions	QS-coated nanodroplets	Properties: Stable HIPEs with 75% oil for over six months of storage. Applications: Formation of transparent oleogels (99.7% oil); Color performance	[31]
Aqueous foams	QS molecules	Properties: Relatively stable foams under pH values 3–5 and salt up to 500 mM NaCl; Reduced rate of Ostwald ripening.	[96]
	QS molecules, food proteins	Properties: Improved foaming properties by proteins β-lactoglobulin and lysozyme.	[26,68,94]
Complex emulsion foams	QS-coated nanodroplets	Properties: Significantly higher foamability and foam stability than QS aqueous foam. Applications: Encapsulation and controlled release of hydrophobic flavors and bioactives.	[98]

**Table 2:** Overview of applications of glycyrrhizic acid (GA) and GA nanofibrils in colloidal multiphase food systems.

Type of Multiphase Systems	Structural Building Blocks	Properties and Applications	Ref.
Liquid O/W emulsions	GA molecules	Properties: Stable emulsions (pH 7.0, 0.2 μm) under pH values 5–9, salts 0–200 mM NaCl, and temperatures up to 60 °C.	[89,90]
	GA nanofibrils	Properties: Emulsions (5 wt% oil, 0.25 wt% nanofibrils) with good stability after repeated heat treatments (80 °C, 20 min) and storage for two months.	[33]
Gel emulsions	GA nanofibrils	Properties: 10–60 wt% oils; High gel strength and thixotropic recovery; Thermoresponsive properties. Applications: Oil structuring materials; Delivery vehicle for oil-soluble ingredients; Green pesticides.	[33,34,91]
	GA nanofibrils, PGPR	Properties: W_1_/O/W_2_ gel emulsions with high yield (85.6–92.5%) and storage stability. Applications: Protection of photosensitive water-soluble cargos (Riboflavin-5′-phosphate).	[78]
	GA nanofibrils, SPI-pectin nanoparticles	Properties: Small droplet size, homogeneous appearance and microstructure at 1 wt% or higher nanofibril concentration.	[88]
	GA nanofibrils, sitosterol–oryzanol mixture	Properties: Dual-structured gel emulsions; Controlled linear and nonlinear viscoelastic behaviors. Applications: Oil structuring materials with specific textural and functional properties.	[92]
	GA nanofibrils, CD–MOF	Properties: Long-term stability, even under high-alkaline pH and high-temperature (70 °C).	[93]
Aqueous foams	GA nanofibrils	Properties: Ultrastable foams with homogeneous appearance for at least six months at 25 °C; Without any liquid drainage; High foamability; Stimulability and processability. Applications: Controlled delivery and release; Solid template for porous materials.	[35]
	GA nanofibrils, CNCs	Properties: Composite foams with higher elastic modulus and yield stress (especially with NaCl); Tunable stability and thermoresponsive behavior.	[79]
Complex emulsion foams	GA nanofibrils	Properties: Stable emulsion foams for at least two weeks; Viscoelastic properties: Thermoresponsive behavior.	[36]
	GA nanofibrils, CBP particles	Properties: Dual photo-/thermoresponsive emulsion foams; On-demand destabilization by multiple external stimuli.	[36]

## 6. Conclusions and Outlook

Driven by the increasing awareness of the importance of health and environmental sustainability, consumers are demanding “clean label” food and beverage products that are formulated with all-natural and sustainable ingredients. Consequently, natural plant-based surfactants and ingredients are highly desired by the food industry to replace many synthetic or animal-based ingredients for reformulating multiphase food products. Due to the strong interfacial activity and many biological activities, saponins are a promising class of natural surfactants and show the great potential for applications in colloidal multiphase food systems. As discussed in this review, the diversity of the amphiphilic structure of the saponins leads to their rich self-assembly behaviors in the aqueous solutions and at the liquid interfaces, which further affect their role in the formation and stabilization of multiphase food systems. Therefore, considering the structural diversity of saponins and its impact on the self-assembly and interfacial properties, this review mainly focuses on the discussion of two commonly used food-grade saponins, Quillaja saponins (QS) and glycyrrhizic acid (GA), with the aim of better clarifying the relationship between structural features of saponin molecules and their subsequent self-assembly, interfacial properties and the applications in multiphase systems.

Compared to the micellar self-assembly of most saponins including QS, the chiral GA molecules exhibit a unique hierarchical self-assembly behavior in water, forming long, semiflexible nanofibrils and supramolecular hydrogels. For their interfacial properties, QS molecules (also many monodesmosidic triterpenoid saponins) can form highly viscoelastic adsorption layers, especially at the air–water interface through multiple hydrogen bonds between the neighboring sugar groups of saponins, whereas GA molecules cannot form a viscoelastic layer, probably due to their very low solubility in water. However, the assembled GA nanofibrils exhibit significantly different behaviors at the air–water and oil–water interfaces. They have a multilayer adsorption at the interfaces, forming dense interfacial fibril networks with a high electrostatic force, which can endow the GA nanofibril-coated emulsion droplets and air bubbles with high stability. These remarkable interfacial properties provide the wide applications of saponins (QS and GA) in the formation and stabilization of various multiphase food systems, including liquid emulsions, gel emulsions, aqueous foams and complex emulsion foams. The emphasis has been on the GA and GA nanofibrils, which are demonstrated to be highly suitable as building blocks for fabricating various multiphase systems with many advanced qualities including simplicity, ultrastability, stimulability, structural viscoelasticity and processability. This is attributed to the spatially controllable assembly of GA nanofibrils at the interfaces and in the continuous phase, forming a combination of the multilayer interfacial network and the viscoelastic continuous hydrogel network. These overall features of GA nanofibril-based multiphase food systems are difficult to achieve from the commonly used food-grade ingredients, such as proteins, polysaccharides, low molecular weight surfactants (including other natural saponins) or their mixtures.

Despite the promising applications of the saponins and the saponin-based multiphase food systems, fundamental studies are still necessary to better understand the underlying mechanism of the formation and stabilization of these complex multiphase systems. For example, further research about the interfacial properties of the saponins with different molecular structures and their relation with the emulsion/foam stabilization is still required. Particularly, more works in the future are expected to explore these new GA nanofibril-based multiphase soft materials that have advanced and multifunctional properties. For instance, considering the excellent stability and smart-responsive properties of the GA nanofibril-based multiphase materials, they can be used as solid delivery vehicles for controlled encapsulation and release of hydrophilic and hydrophobic bioactives or flavor components. GA nanofibrils also have the ability as effective structuring agents to create soft-solid gel emulsions for edible oil structuring. In addition, the GA nanofibril-based multiphase colloids have outstanding stability and are highly viscoelastic and yield stress materials, suggesting the great potential as edible inks in three-dimensional (3D) food printing. These natural saponin-based multiphase systems are expected to meet the demands as sustainable, plant-based ingredients for more sustainable applications in the fields of future foods, cosmetics and pharmaceutics.

## Data Availability

Not applicable.
